# The Impact of Aging and Toll-like Receptor 2 Deficiency on the Clinical Outcomes of *Staphylococcus aureus* Bacteremia

**DOI:** 10.1093/infdis/jiad046

**Published:** 2023-02-20

**Authors:** Zhicheng Hu, Pradeep Kumar Kopparapu, Meghshree Deshmukh, Anders Jarneborn, Priti Gupta, Abukar Ali, Ying Fei, Cecilia Engdahl, Rille Pullerits, Majd Mohammad, Tao Jin

**Affiliations:** Department of Rheumatology and Inflammation Research, Institute of Medicine, Sahlgrenska Academy, University of Gothenburg, Gothenburg, Sweden; Center for Clinical Laboratories, the Affiliated Hospital of Guizhou Medical University, Guiyang, China; Department of Rheumatology and Inflammation Research, Institute of Medicine, Sahlgrenska Academy, University of Gothenburg, Gothenburg, Sweden; Department of Rheumatology and Inflammation Research, Institute of Medicine, Sahlgrenska Academy, University of Gothenburg, Gothenburg, Sweden; Department of Rheumatology and Inflammation Research, Institute of Medicine, Sahlgrenska Academy, University of Gothenburg, Gothenburg, Sweden; Department of Rheumatology, Sahlgrenska University Hospital, Gothenburg, Sweden; Department of Rheumatology and Inflammation Research, Institute of Medicine, Sahlgrenska Academy, University of Gothenburg, Gothenburg, Sweden; Department of Rheumatology and Inflammation Research, Institute of Medicine, Sahlgrenska Academy, University of Gothenburg, Gothenburg, Sweden; Center for Clinical Laboratories, the Affiliated Hospital of Guizhou Medical University, Guiyang, China; Department of Rheumatology and Inflammation Research, Institute of Medicine, Sahlgrenska Academy, University of Gothenburg, Gothenburg, Sweden; Department of Rheumatology and Inflammation Research, Institute of Medicine, Sahlgrenska Academy, University of Gothenburg, Gothenburg, Sweden; Department of Clinical Immunology and Transfusion Medicine, Sahlgrenska University Hospital, Gothenburg, Sweden; Department of Rheumatology and Inflammation Research, Institute of Medicine, Sahlgrenska Academy, University of Gothenburg, Gothenburg, Sweden; Department of Rheumatology and Inflammation Research, Institute of Medicine, Sahlgrenska Academy, University of Gothenburg, Gothenburg, Sweden; Department of Rheumatology, Sahlgrenska University Hospital, Gothenburg, Sweden

**Keywords:** *Staphylococcus aureus*, TLR2, aging, bacteremia, mouse

## Abstract

*Staphylococcus aureus* (*S. aureus*) causes a broad range of infections. Toll-like receptor (TLR) 2 senses the *S. aureus* lipoproteins in *S. aureus* infections. Aging raises the risk of infection. Our aim was to understand how aging and TLR2 affect the clinical outcomes of *S. aureus* bacteremia. Four groups of mice (wild type/young, wild type/old, TLR2^−/−^/young, and TLR2^−/−^/old) were intravenously infected with *S. aureus*, and the infection course was followed. Both TLR2 deficiency and aging enhanced the susceptibility to disease. Increased age was the main contributing factor for increased mortality rates and changes in spleen weight, whereas other clinical parameters, such as weight loss and kidney abscess formation, were more TLR2 dependent. Importantly, aging increased mortality rates without relying on TLR2. In vitro, both aging and TLR2 deficiency down-regulated cytokine/chemokine production of immune cells with distinct patterns. In summary, we demonstrate that aging and TLR2 deficiency impair the immune response to *S. aureus* bacteremia in distinct ways.

## Teaser

In Staphylococus aureus bacteremia mouse model, aging and TLR2 deficiency impair the immune responses in distinct patterns. Mortality was more controlled by aging, whereas weight loss and kidney abscess were more TLR2 dependent. Aging increased mortality independent of TLR2 expression.

*Staphylococcus aureus* (*S. aureus*) bacteremia is associated with higher mortality rates (10%–30%) than bacteremia caused by most other microbes [1, 2]. *S. aureus* sepsis is caused by bacterial components and an exaggerated immune response mounted by the host [3]. *S. aureus* lipoproteins are the most potent proinflammatory bacterial components that significantly contribute to the pathogenesis of sepsis by stimulating a rapid release of chemokines and cytokines through Toll-like receptor (TLR) 2 [4-6]. TLR2-deficient (TLR2^−/−^) mice, lacking staphylococcal lipoprotein ligand recognition, are known to be more sensitive to *S. aureus* systemic infections and associated with an increased mortality rate [7].

Human life expectancy in developed countries has increased dramatically over the past century and will continue to rise in the future [8]. Aging causes immune system dysfunction, which may lead to an increased risk of infections. In innate immunity, the phagocytic function of human neutrophils is impaired in the older population [9]. Similarly, aged mice are unable to mount an effective innate immune response to polymicrobial sepsis [10]. In adaptive immunity, the proportion of naive T cells is down-regulated in older persons, which results in decreased repertoire diversity to foreign antigens and reduces the antibody response to infection [11]. Indeed, the incidence for severe sepsis increases drastically with age [12-14]. The mortality rate of sepsis also increases with age, from 10% in children to 38.4% in adults aged ≥85 years [14]. Age-associated alterations in TLR function have been linked to the increased morality rate [15]. For instance, the production of tumor necrosis factor (TNF) α and interleukin-6 (IL-6), after TLR1/2 engagement, was down-regulated in older adults, compared with younger populations [16], suggesting that diminished TLR1/2 signaling contributes to an increased risk of infection in older adults.

It is still largely unknown how aging affects the clinical outcomes of *S. aureus* bacteremia. Moreover, because TLR2 senses the *S. aureus* lipoprotein and plays a potent role in *S. aureus* infections, it may contribute to sepsis pathogenesis in the context of aging. To answer these questions, we used our well-established *S. aureus* bacteremia model with old and young as well as TLR2-knockout mice. Our data show that both aging and TLR2 deficiency impair the immune responses to *S. aureus* bacteremia in distinct patterns. Interestingly, aging increases mortality rates independently of TLR2.

## METHODS

### Ethics Statement

Mouse studies were reviewed and approved by the Ethics Committee of Animal Research of Gothenburg and conducted in accordance with the regulations and recommendations of the Swedish Board of Agriculture for animal experiments.

### Mice

C57BL/6 wild-type (WT) mice and TLR2^−/−^ B6.129-Tlr2^tm1Kir^/J mice of both sexes were purchased from the Jackson Laboratory (Bar Harbor, Maine). All mice were housed and bred until they reached the desired age range as young (13–28 weeks old) or old (73–89 weeks old).

### Preparation of Bacterial Solutions

*S. aureus* (Newman WT) strain was prepared as described elsewhere [17]. For heat-killed bacteria, *S. aureus* strain was heat killed at 95°C for 45 minutes [18].

### Experimental Protocols for *S. aureus* Bacteremia Mouse Model

The mouse bacteremia model was performed as described elsewhere [19]. Briefly, 200 µL of *S. aureus* suspension (1.5 × 10^6^ colony-forming units [CFUs] per mouse) was inoculated intravenously into the tail vein of mice. The animals were monitored on a daily basis by 3 observers (Z. H., M. M., and T. J.) for up to 10 days after infection. To estimate the severity of arthritis, a clinical scoring system ranging from 0 to 3 was used, as described elsewhere [20, 21]. Surviving mice were euthanized on day 10 after infection, the blood samples, kidneys, spleens, and paws were collected. The data were pooled from 2 independent experiments. For each experiment, we included all 4 groups with similar group size and sex distribution (Supplementary Table 1).

### Bacteriological Examination of Kidneys and Joints

Kidney abscess scores were assessed by 2 investigators (Z. H. and M. M.) in a blinded manner [22]. The scoring system used ranged from 0 to 3 (0 indicates healthy kidneys; 1, 1–2 small abscesses in the kidneys without structure changes; 2, >2 abscesses, but <75% kidney tissue involved; and 3, large amounts of abscess with >75% kidney tissue involved). The kidneys were then homogenized and plated on horse blood agar plates to quantify the CFU counts.

### Flow Cytometry

Sample processing and flow cytometric analysis of mouse blood cells were performed as described elsewhere [23]. Briefly, an ethylenediaminetetraacetic acid–coated tube was used to collect mouse peripheral blood. Red blood cell lysis was performed by adding eBioscience 1X RBC Lysis Buffer (Invitrogen). The cells were blocked with Mouse BD FcBlock (BD Biosciences) and labeled with antibody cocktail (Supplementary Table 2). Sample acquisition was performed using BD FACSLyric flow cytometry (BD Biosciences), and data were analyzed using FlowJo software (version 10.8; Tree Star).

### Micro–Computed Tomography

All 4 paws were scanned with a SkyScan 1176 micro–computed tomographic scanner (Bruker). NRecon software (version 1.6.9.8; Bruker) was used to reconstruct 3-dimensional images, and CTvox software (version 2.7.0; Bruker) was subsequently used for evaluation. Each joint was evaluated using a scoring system, as described elsewhere [21].

### In Vitro Splenocyte and Peritoneal Macrophage Stimulation

The procedure was adapted as described elsewhere [4]. The spleens were aseptically removed and passed through a cell strainer (70-μm nylon mesh). Peritoneal lavage was performed to collect the peritoneal cells by using 10 mL of ice-cold phosphate-buffered saline. Ammonium chloride (0.83%) was used to lyse the erythrocytes. The cell suspensions were grown in Iscove's complete medium with 10% fetal calf serum (Sigma-Aldrich). Both splenocytes and peritoneal cells were incubated with purified lipopolysaccharide (LPS; 1 μg/mL), Pam3CSK4 (20 ng/mL), toxic shock syndrome toxin (TSST) 1 (100 ng/mL), heat-killed *S. aureus* Newman WT strain (1 × 10^7^ CFUs/mL), or Iscove's medium (negative control).

### Measurement of Cytokine/Chemokine Levels

The levels of IL-6, TNF-α, macrophage inflammatory protein (MIP) 2, and keratinocyte chemoattractant (KC) in the blood or cell cultures were quantified using DuoSet ELISA kits (R&D Systems Europe), according to the manufacturer's instructions.

### Chemicals

Isoluminol was purchased from Sigma-Aldrich, phorbol 12-myristate 13-acetate was purchased from MilliporeSigma, phenol-soluble modulin α3 was purchased from EMC, and *N*-formyl-Met-Ile-Phe-Leu (fMIFL) was synthesized by TAG Copenhagen.

### Isolation of Mouse Neutrophils

Distal tips of the femur and tibia were cut. Bone marrow cells were collected by forcing ice cold Krebs-Ringer glucose phosphate buffer through the bone marrow cavities. Residual erythrocytes were removed by hypotonic lysis. Mouse neutrophils were isolated following the standard procedure of discontinuous Percoll (GE Healthcare) density gradient centrifugation.

### Measurements of NADPH Oxidase Activity

The production of superoxide anion by the neutrophil reduced nicotinamide adenine dinucleotide phosphate hydrogen (NADPH) oxidase was measured using a isoluminol-amplified chemiluminescence technique performed by a 6-channel Biolumat LB 9505 luminometer (Berthold), as described elsewhere [24].

### Statistical Analysis

Statistical analyses were performed using GraphPad Prism software, version 9 (GraphPad Software). Comparisons among experimental groups were assessed using the log-rank (Mantel-Cox) test, ANOVA with Tukey's test, or Kruskal-Wallis test with Dunn's posttest, as appropriate. All results are reported as the median or mean with standard error of the mean, unless otherwise indicated. Differences were considered statistically significant at *P* < .05.

## RESULTS

### Impacts of Aging and TLR2 on the Outcome of *S. aureus* Bacteremia in Mice

**Figure 1.**
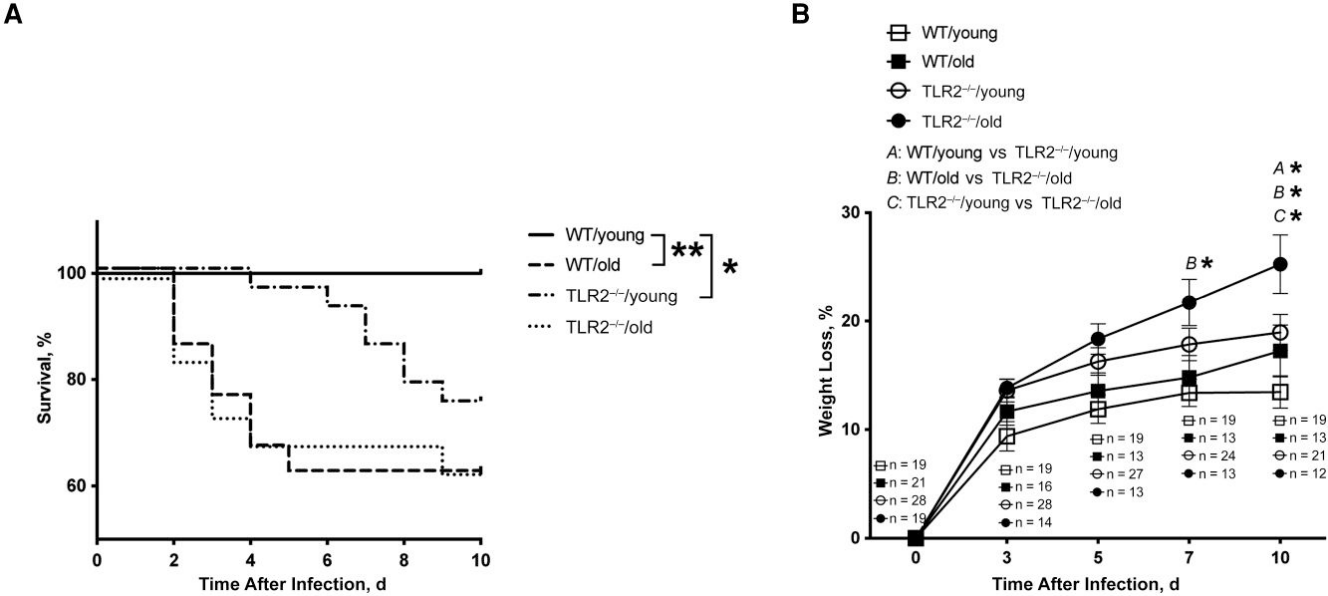
Aging and Toll-like receptor (TLR) 2 had a significant impact on the outcome of *Staphylococcus aureus* bacteremia in mice. C57BL/6 wild-type (WT) mice and TLR2–deficient (TLR2^−/−^) mice of both sexes, aged 13–28 weeks (young) or 73–89 weeks (old), were intravenously inoculated with the *S. aureus* Newman strain at a dose of 1.5 × 10^6^ colony-forming units per mouse. The surviving mice were euthanized on day 10 after infection. Cumulative survival was followed up every 12 hours (*A*), and the changes in body weight were monitored up to 10 days after infection (*B*). The data were pooled from 2 independent experiments. Statistical evaluations were performed using the log-rank (Mantel-Cox) test (*A*) or 2-way ANOVA with Tukey's test (*B*). Data in *B* are presented as means with standard errors of the mean. *P < .05; **P < .01.

C57BL/6 WT mice and TLR2^−/−^ mice of both young and old age groups were intravenously injected with the *S. aureus* Newman strain. The overall mortality rate was significantly higher in both the TLR2^−/−^/old and WT/old mice than in the WT/young group. Death occurred much sooner after infection in aged mice than in young mice (Figure 1*A*). At the end of the experiment, the survival rate among the WT/old group was 62%, compared with 100% in the WT/young group (*P* = .003). TLR2 deficiency also increased the mortality rate in young mice, of which 75% survived (*P* = .02). Intriguingly, aging played a dominant role in sepsis deaths compared with TLR2, as WT/old and TLR2^−/−^/old mice both had similar mortality rates.

The greatest weight loss was found in aged TLR2^−/−^ mice (Figure 1*B*). Overall, compared with WT mice, TLR2^−/−^ mice showed significantly more pronounced weight reduction on days 7 and 10 after infection. Regarding the age factor, the only significant difference was observed between TLR2^−/−^/young and TLR2^−/−^/old mice on day 10 after infection. Our data indicate that, independent of TLR2, aging leads to increased mortality rates, whereas weight development is largely dependent on TLR2.

### TLR2 as Determinant of Bacterial Clearance in Kidneys

**Figure 2.**
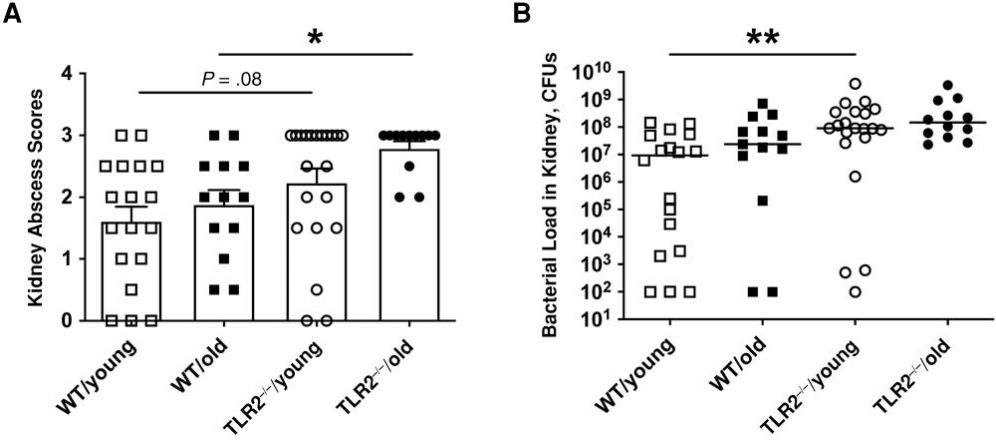
Toll-like receptor (TLR) 2, rather than aging, determined bacterial clearance in kidneys. C57BL/6 wild-type (WT) mice and TLR2-deficient (TLR2^−/−^) mice of both sexes, aged 13–28 weeks (young) or 73–89 weeks (old)—including WT/young (n = 19), WT/old (n = 21), TLR2^−/−^/young (n = 28) and TLR2^−/−^/old (n = 19) groups—were intravenously inoculated with the *Staphylococcus aureus* Newman WT strain at a dose of 1.5 × 10^6^ colony-forming units (CFUs) per mouse. The surviving mice were euthanized on day 10 after infection. *A,* Kidney abscess scores. *B,* Persistence of *S. aureus* in kidneys 10 days after infection. The data were pooled from 2 independent experiments. Statistical evaluations were performed using Kruskal-Wallis test with Dunn's posttest. Data are presented as means with standard errors of the mean (*A*) or medians (*B*). *P < .05; **P < .01.

Bacterial load is an important parameter reflecting the host bacterial clearance capacity. More abscesses were noticed on the kidneys of TLR2^−/−^ mice irrespective of age on day 10 after infection (Figure 2*A*). Similarly, higher bacterial counts were found in the kidneys of TLR2^−/−^ young mice than in the WT groups (Figure 2*B*), indicating that TLR2 deficiency impairs the bacterial clearance capacity of the host independently of age.

### Spleen Weight Changes and Cytokine Profiles in Aged Mice With Staphylococcal Bacteremia

**Figure 3.**
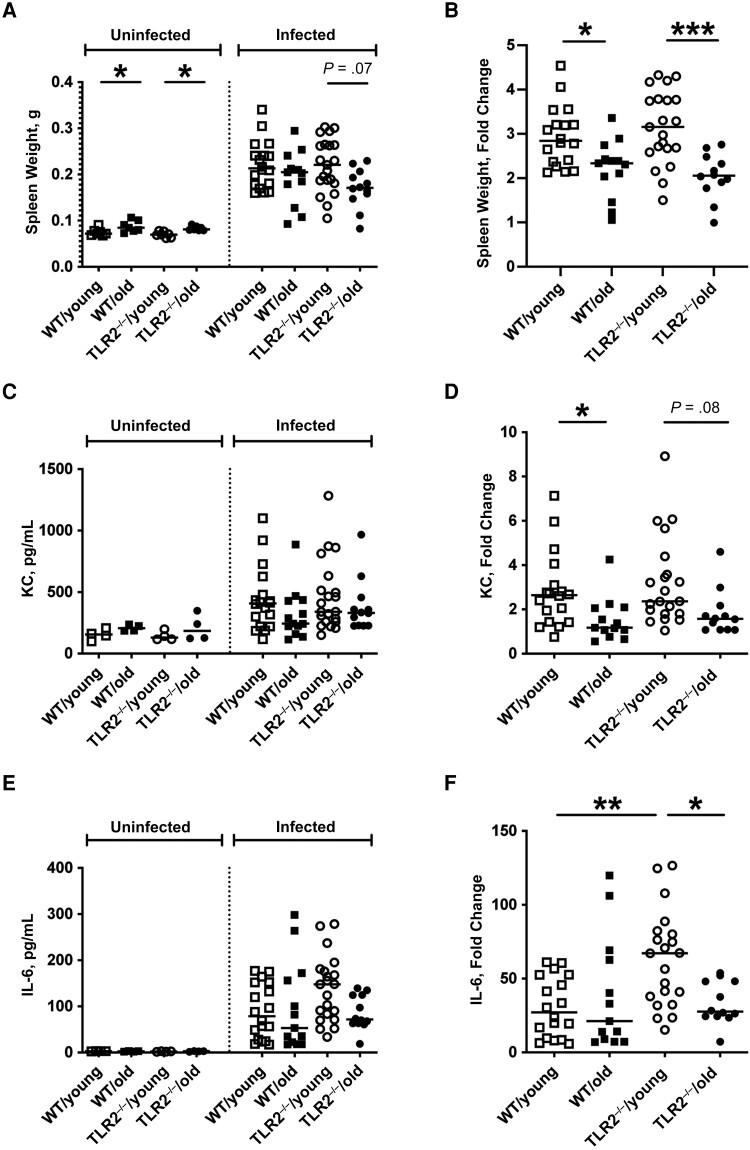
Spleen weight changes and cytokine profiles were affected in aged mice with staphylococcal bacteremia. C57BL/6 wild-type (WT) mice and Toll-like receptor (TLR) 2–deficient (TLR2^−/−^) mice of both sexes, aged 13–28 weeks (young) or 73–89 weeks (old)—including WT/young (n = 19), WT/old (n = 21), TLR2^−/−^/young (n = 28), and TLR2^−/−^/old (n = 19) groups—were intravenously inoculated with the *Staphylococcus aureus* Newman WT strain at a dose of 1.5 × 10^6^ colony-forming units per mouse. Surviving mice were euthanized on day 10 after infection. *A, C, E,* Spleen weight (*A*), peripheral blood levels of keratinocyte-derived chemokine (KC) (*C*), and interleukin 6 (IL-6) (*E*) were analyzed 10 days after infection and in respective groups of healthy (uninfected) mice (n = 7 [*A*] or n = 4 [*C, E*] per group). *B, D, F,* Fold changes in spleen weight (*B*), KC levels (*D*), and IL-6 levels (*F*) compared with healthy mice. Data were pooled from 2 independent experiments. Statistical evaluations were performed using 1-way ANOVA with Tukey's test or Kruskal-Wallis test with Dunn's posttest. Lines in dot plots represent medians. *P < .05; **P < .01; ***P < .001.

Spleen enlargement in an infection indicates the functional immune response from splenocytes against pathogens [25]. We found that aged mice had significantly enlarged spleens than the young mice before infection (Figure 3*A*). Thus, to roughly understand how aging and TLR deficiency affect the immune responses, the fold changes of spleen weight after infection were calculated based on the average spleen weight from respective groups of healthy control mice (Supplementary Table 3). All groups had splenomegaly on day 10 after infection (Figure 3*A*). Older mice had significantly lower fold changes of spleen weight than young mice irrespective of TLR2 expression (Figure 3*B*), suggesting that the capacity of splenocyte proliferation in response to *S. aureus* bacteremia is mainly dependent on age.

We further measured the cytokine/chemokine levels in these 4 groups of mice before and after infection (Figure 3*C* and 3*E*). Both KC and IL-6 serum levels were increased after infection. The fold changes of cytokine levels after infection were calculated based on the average levels from respective groups of healthy control mice (Supplementary Table 4). In line with the data from spleen weight changes, fold changes of KC levels were lower in aged mice than in young mice, irrespective of TLR2 expression (Figure 3*C* and *D*). However, lower fold changes of IL-6 levels in aged mice were found only in the TLR2^−/−^ mice, and the TLR2^−/−^/young group had the highest fold changes among all the groups (Figure 3*E* and 3*F*). We also measured TNF-α and MIP-2 serum levels, which were undetectable on day 10 after infection.

### Impact of Sex Differences on Outcomes of *S. aureus* Bacteremia

To further understand whether sex contributed to the differences observed, the outcomes of *S. aureus* bacteremia were compared between male and female mice in all 4 groups. No difference was observed between sexes in 4 groups regarding mortality rates, kidney abscesses, and kidney CFU counts (Supplementary Figures 2 and 3). Regarding weight loss, a significant difference was found only in the TLR2^−/−^/old group, in which male mice had lost significantly more weight than female mice on day 10 after infection (Supplementary Figure 1).

### Changes in Blood Leukocyte Population After Staphylococcal Bacteremia in Aged TLR2^−/−^ Mice

**Figure 4.**
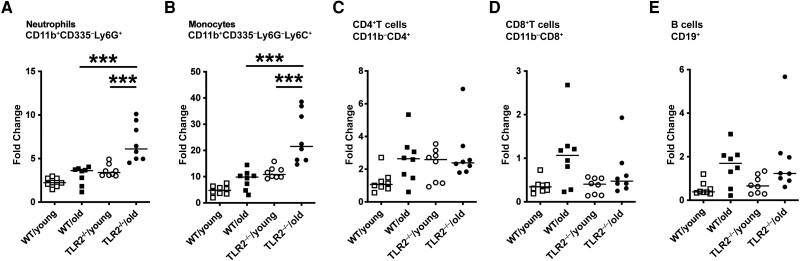
Leukocyte population changed in blood after staphylococcal bacteremia in aged Toll-like receptor (TLR) 2–deficient (TLR2^−/−^) mice. C57BL/6 wild-type (WT) mice and TLR2^−/−^ mice of both sexes, aged 14–17 weeks (young) or 74–76 weeks (old), were intravenously inoculated with *Staphylococcus aureus* Newman WT strain at a dose of 1.5 × 10^6^ colony-forming units per mouse. Mice were euthanized, and blood samples (n = 8 per group) were collected for flow cytometric analyses on day 10 after infection. Fold changes in different cell populations were calculated based on the average reference from a group of healthy mice. The cell populations were gated as followed: neutrophils, CD11b^+^CD335^−^Ly6G^+^ (*A*); monocytes, CD11b^+^CD335^−^Ly6G^−^Ly6C^+^ (*B*); CD4^+^ T cells, CD11b^−^CD4^+^ (*C*); CD8^+^ T cells, CD11b^−^CD8^+^ (*D*); and B cells, CD19^+^ (*E*). Statistical evaluations were performed using 1-way ANOVA with Tukey's test, and lines indicate medians. ***P < .001.

To understand the cellular change in different mice groups after staphylococcal bacteremia, we used flow cytometry to further investigate the peripheral leukocyte population on day 10 after infection. The fold changes in leukocyte populations after infection were calculated based on the average cell frequencies from respective groups of healthy control mice (Supplementary Table 5). The highest fold changes in neutrophils (Figure 4*A*) and monocytes (Figure 4*B*) among the groups were found in TLR2^−/−^/old mice, and the lowest changes were observed in the WT/young group. No significant difference was found among groups regarding adaptive immune responses, including fold changes of B and T cells (Figure 4*C*–4*E*).

### Down-regulation of In Vitro Splenocyte TNF Production in Aged Mice

**Figure 5.**
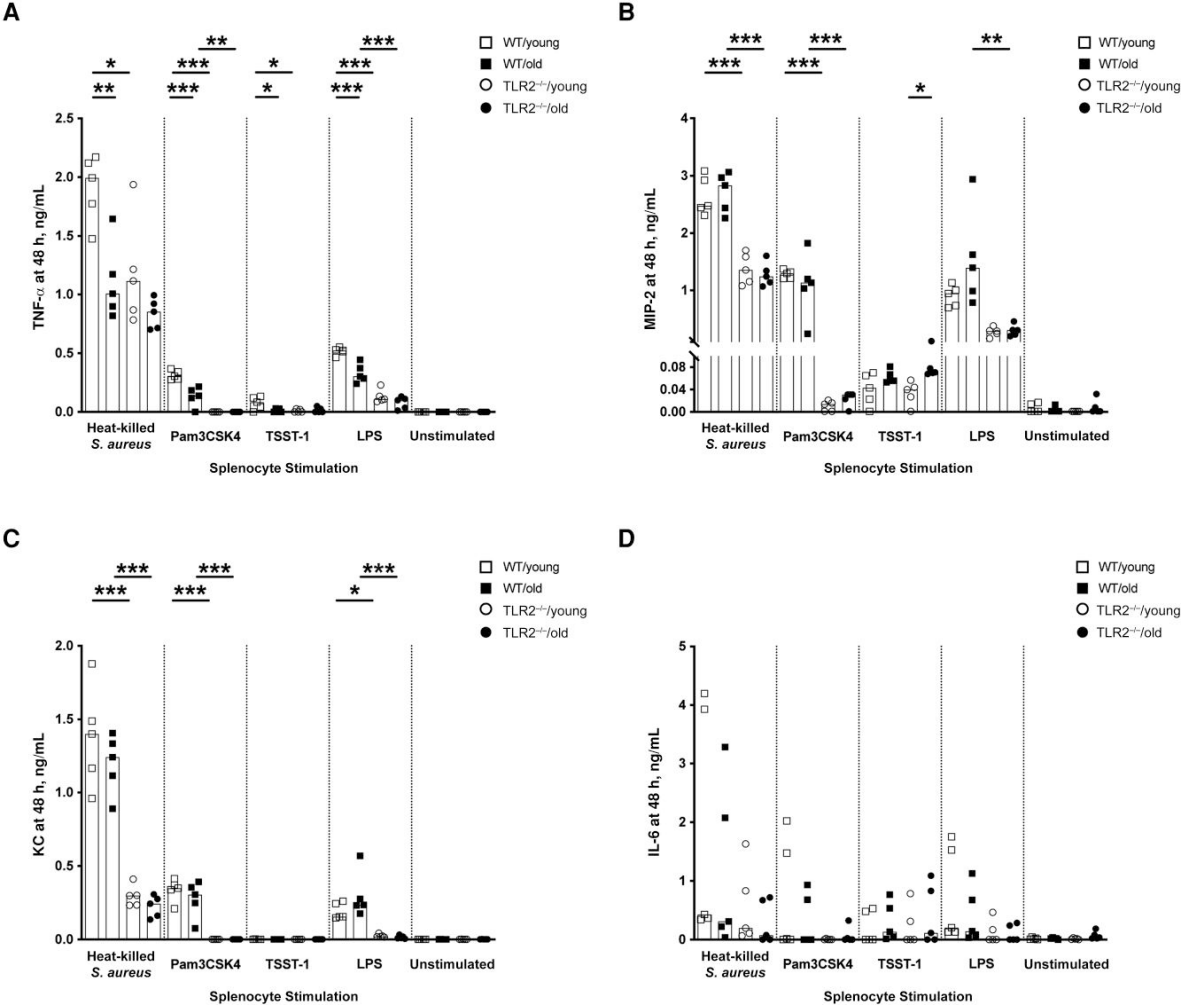
In vitro splenocyte tumor necrosis factor (TNF) production was down-regulated in aged mice. *A–D,* Levels of TNF-α (*A*), macrophage inflammatory protein (MIP) 2 (*B*), keratinocyte chemoattractant (KC) (*C*), and interleukin 6 (IL-6) (*D*) in the supernatants from splenocyte cultures (1 × 10^6^ cells/mL) collected from young (aged 13–16 weeks) and old (aged 75–82 weeks) C57BL/6 wild-type (WT) and Toll-like receptor (TLR) 2–deficient (TLR2^−/−^) mice (n = 5 per group) after 48 hours of stimulation with heat-killed *Staphylococcus aureus* (Newman WT strain, 1 × 10^7^ colony-forming units/mL), Pam3CSK4 (20 ng/mL), toxic shock syndrome toxin 1 (TSST-1; 100 ng/mL), or purified lipopolysaccharide (LPS) (1 µg/mL). Data were pooled from 2 independent experiments. Statistical evaluations were performed using 1-way ANOVA with Tukey's test, and bars indicate median values. *P < .05; **P < .01; ***P < .001.

Splenocytes collected from all 4 groups of mice were stimulated with heat-killed *S. aureus*, Pam3CSK4, TSST1, or LPS. TNF-α production was lower in both aged and TLR2^−/−^ mice compared with WT/young mice when heat-killed *S. aureus*, Pam3CSK4, and LPS were used (Figure 5*A*). In contrast, neutrophil chemoattractant (MIP-2 and KC) release was more TLR2 dependent, as the levels of MIP-2 and KC were lower in TLR2^−/−^ than in WT groups, irrespective of age (Figure 5*B* and 5*C*). No tangible difference was found in IL-6 expression (Figure 5*D*). These data suggest that both aging and TLR2 control TNF production, whereas TLR2, but not aging, regulates MIP-2/KC production when splenocytes are stimulated with *S. aureus* components.

### Age and TLR2 Dependence of In Vitro IL-6, MIP-2, and KC Production by Peritoneal Macrophages

**Figure 6.**
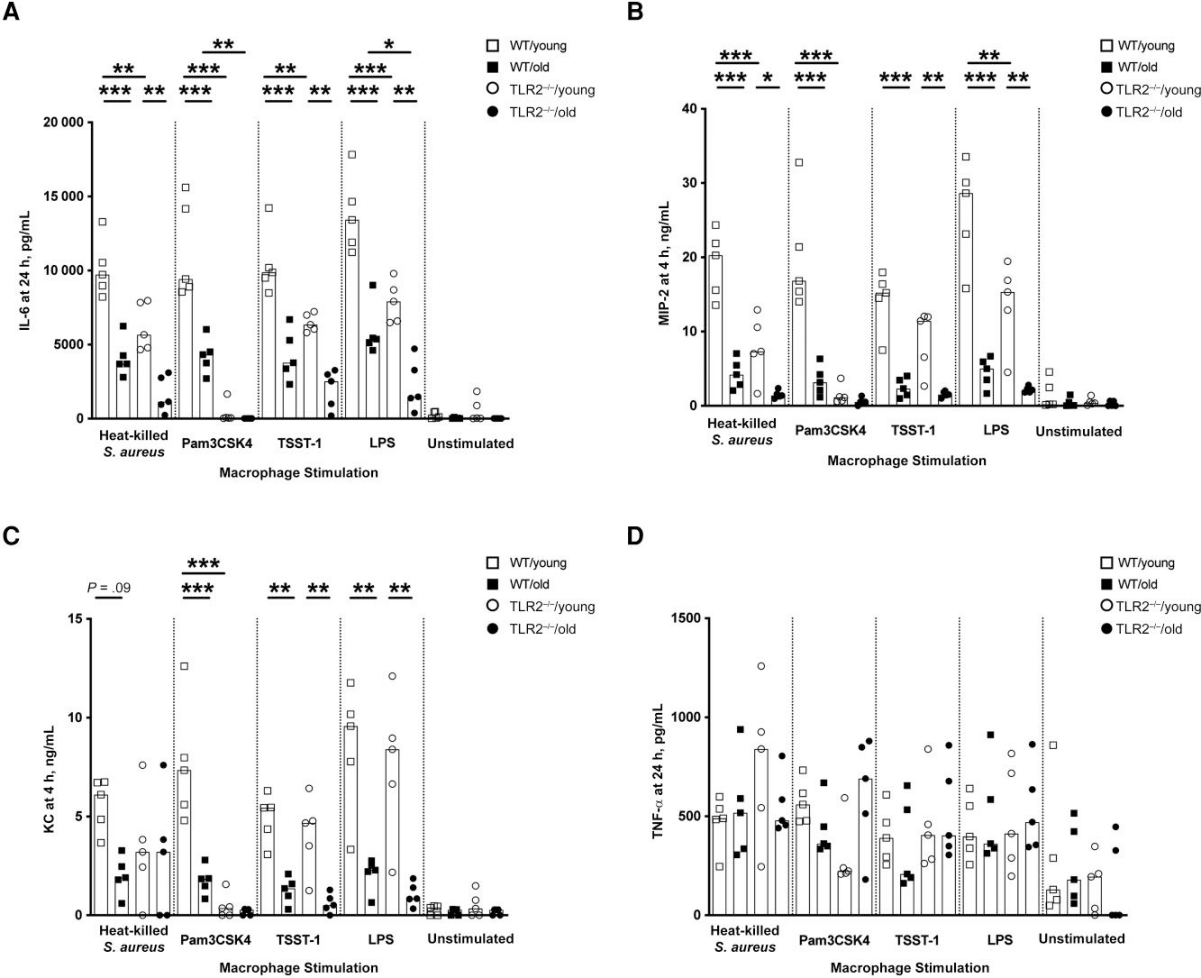
In vitro interleukin 6 (IL-6), macrophage inflammatory protein (MIP) 2, and keratinocyte chemoattractant (KC) production by peritoneal macrophages was age and Toll-like receptor (TLR) 2 dependent. *A–D,* Levels of IL-6 (*A*), MIP-2 (*B*), KC (*C*), and tumor necrosis factor (TNF) α (*D*) in the supernatants from peritoneal cell cultures (1 × 10^6^ cells/mL) collected from C57BL/6 wild-type (WT) and TLR2-deficient (TLR2^−/−^) mice of both sexes, aged 13–16 weeks (young) or 75–82 weeks (old) (n = 5 per group) after stimulation with heat-killed *Staphylococcus aureus* (Newman WT strain, 1 × 10^7^ colony-forming units/mL), Pam3CSK4 (20 ng/mL), toxic shock syndrome toxin 1 (TSST-1; 100 ng/mL), or purified lipopolysaccharide (LPS; 1 µg/mL) for 4 or 24 hours. Data were pooled from 2 independent experiments. Statistical evaluations were performed using 1-way ANOVA with Tukey's test, and data are presented as medians. *P < .05; **P < .01; ***P < .001.

Significant differences were observed between WT and TLR2^−/−^ groups and between young and old groups (Figure 6*A*), indicating that the IL-6 release induced by *S. aureus* infection depends not only on age but also on TLR2. In line with IL-6, MIP-2 and KC also showed a rapid release in the supernatant of peritoneal cells collected from WT/young mice after 4 hours of stimulation (Figure 6*B* and 6*C*). The cells collected from aged mouse groups exhibited the lowest levels of MIP-2 and KC. This effect was mainly dependent on age but partially relied on TLR2, as differences between WT groups and TLR2^−/−^ groups were found for MIP-2 with heat-killed *S. aureus*, Pam3CSK4, and LPS and for KC with Pam3CSK4 only. In contrast to the significantly lower TNF-α release in the splenocyte cultures of aged mice, there was no difference among the groups for TNF-α production in peritoneal macrophage cell cultures (Figure 6*D*).

### Age and TLR2 and the Severity of *S. aureus*–Induced Septic Arthritis

We also compared the severity and frequency of clinical arthritis among those 4 groups of mice. No significant differences between groups were observed in terms of arthritis severity and frequency during the course of infection, regardless of age or TLR2 (Supplementary Figure 4). Surprisingly, the WT/young group had the highest frequency of clinical arthritis (47%) among the groups on day 10 after infection, while the TLR2^−/−^/old group had the lowest frequency (23%) (Supplementary Figure 4*B*). Micro­–computed tomography was used to evaluate the destruction of mouse joints collected on day 10 after infection. Similar severity and frequency of bone erosions were observed among groups (Supplementary Figures 5*A* and 5*B*).

### TLR2 and Aging and Mouse Neutrophil NADPH Oxidase Release

To understand the impact of aging and TLR2 deficiency on the neutrophil activity, we measured the NADPH oxidase release level after mouse neutrophils were stimulated by formylated tetrapeptide fMIFL (formyl peptide receptor [FPR] 1 agonist), phenol-soluble modulin α3 (FPR2 agonist [23]) or phorbol 12-myristate 13-acetate (protein kinase C agonist [26]), respectively. No tangible differences were found among groups (Supplementary Figures 6*A*–6*F*). These data indicate that the capacity of mouse neutrophil NADPH oxidase release relies on neither age nor TLR2.

## DISCUSSION

Our data strongly suggest that both age and TLR2 are crucial host factors in *S. aureus* bacteremia. To our surprise, it seems that aging has an even greater impact on bacteremia mortality rates than the lack of TLR2. In general, aging and TLR2 deficiency aggravate the disease in an additive manner in terms of weight loss and formation of kidney abscesses. However, the combination of increased age and TLR2 deficiency did not further increase mortality rates; these were similar in aged WT and aged TLR2 mice, suggesting that the TLR2-related functions are probably impaired in aged WT mice.

It has been shown that TLR2/TLR1- but not TLR2/TLR6-induced cytokine production was impaired in older adults compared with young controls. Baseline surface expression was decreased in the older adults for TLR1 but not TLR2, suggesting age-associated defect in TLR2/TLR1 function [16]. Indeed, it has been shown that the early production of proinflammatory cytokines, such as IL-6 and TNF-α, in alveolar macrophages was reduced with age in a murine pneumococcal pneumonia model, and the reduced cytokine production was due to age-related impairment in the TLR2 signaling pathway rather than expression of TLRs [27]. In another study, aging has also been shown to reduce TLR2 signaling without altering TLR2 expression in mouse macrophages [28].

There are discrepancies in mortality rates and weight loss amount among the 4 groups of mice. Mortality rates were dependent on both age and TLR2; they were significantly higher in both WT/old and TLR2^−/−^/young mice than in WT/young mice and also tended to be higher in TLR2^−/−^/old mice than in TLR2^−/−^/young mice. In contrast, weight loss during *S. aureus* bacteremia seemed to be mostly TLR2 dependent. It is most likely that aging determines lethality in bacteremia but TLR2 affects both mortality rates and weight loss. However, the discrepancy might also be explained by the fact that more of the TLR2^−/−^/old and WT/old mice were deceased, as deceased mice usually had more severe disease and lost more weight.

TLR2 deficiency causes the overgrowth of *S. aureus* in the kidneys during bacteremia [6, 7]. Our previous studies demonstrated that weight loss correlates well with the bacterial load in kidneys in mice with *S. aureus* bacteremia [7]. Indeed, in the current study, weight loss was also TLR2 dependent, just as kidney abscesses and bacterial counts were. IL-6 mediates fever and food intake in mice with sepsis [29]. IL-6 levels were positively correlated with kidney bacterial counts in this animal model [21]. In line with previous findings, we showed higher fold changes in IL-6 levels after infection in TLR2^−/−^/young mice compared with WT/young mice. Intriguingly, in TLR2^−/−^/old mice, the most severely sick group with the highest weight loss and bacterial persistence in the kidneys, displayed a weak IL-6 response to infection, suggesting that advanced age has a great impact on IL-6 production. In addition, the fold change of KC levels in blood was less pronounced in older than in younger mice, irrespective of TLR2 expression.

The cytokine/chemokine responses were perfectly in line with the fold changes in spleen weight in response to *S. aureus* bacteremia, as the spleen weight changes were significantly reduced in aged mice compared with young mice in both WT and TLR2^−/−^ groups. Simultaneously, we should bear in mind that differences in bacterial load in kidneys in the late phase of infection (day 10 after infection) may drive some differences in immune responses, such as cytokine/chemokine production, and therefore the cytokine levels on day 10 after infection may not directly reflect TLR2- or age-specific effects. Future studies are warranted to clarify cytokine/chemokine responses in the early phase of infection (day 2–3 after infection).

In vitro stimulation experiments displayed similar data, as both aging and TLR2 deficiency down-regulated cytokine and chemokine production from both splenocytes and macrophages in response to stimulation of *S. aureus* components, such as heat-killed *S. aureus* and lipopeptide Pam3CSK4. TNF-α is crucial to the hypothermia and lethality associated with sepsis in mice [29]. Anti-TNF treatment was shown to prevent lethal septic shock in gram-negative bacteremia [30] and staphylococcal enterotoxin shock syndrome, as well as in *S. aureus* sepsis [20]. Simultaneously, TNF-α is a potent cytokine that maintains the T-helper 1 immune response that is important for the elimination of invaders. Elevated serum TNF levels were an independent prognostic marker for all-cause mortality in persons aged 100 years [31]. The baseline production of cytokines was increased in older compared with healthy young persons [32-34]. However, impaired production of TNF-α and interleukin 1 in whole blood was observed following in vitro LPS stimulation in older persons compared with young controls [35]. We speculate that impaired cytokine production likely contributes to the high mortality rates in aged mice.

The myeloid responses (neutrophil and monocyte fold changes) after *S. aureus* bacteremia were increased in TLR2^−/−^ mice, which might be just the host response to a higher bacterial load in organs due to TLR2 deficiency. Interestingly, in WT mice, there was no increase or only a minor increase in myeloid leukocytes in old compared with the young mice. However, in the context of TLR2 deficiency, the fold increases in both neutrophils and monocytes were much larger in old than in young mice, suggesting that older mice probably maintain the normal function of bone marrow granulopoiesis and myelopoiesis and that TLR2 expression suppresses hematopoiesis, especially in aged mice. Indeed, it has been shown before that splenic and bone marrow myeloid cells in septic aged mice exhibited decreased phagocytosis, reactive oxygen species production, and chemotaxis despite increased cell numbers in circulation [10].

**Figure 7.**
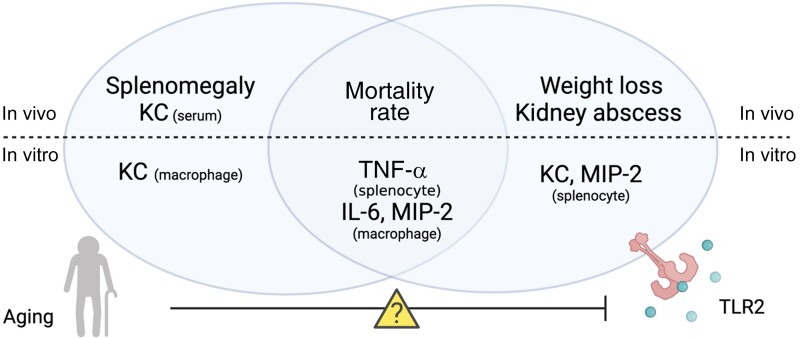
The role of aging and Toll-like receptor (TLR) 2 in *Staphylococcus aureus* bacteremia in mice. The splenomegaly and keratinocyte chemoattractant (KC) production in *S. aureus* bacteremia are more controlled by aging, whereas other clinical parameters such as weight loss and kidney abscess are more TLR2 dependent. Both aging and TLR2 deficiency impair the cytokine/chemokine production in vitro splenocyte/macrophage stimulation. Both TLR2 and aging control the mortality rate associated with *S. aureus* bacteremia. However, the combination of increased age and TLR2 deficiency did not further increase the mortality rate, suggesting that the TLR2-related functions might be impaired in older individuals, which warrant future mechanism studies. Abbreviations: IL-6, interleukin 6; MIP, macrophage inflammatory protein; TNF, tumor necrosis factor.

Our data demonstrated a subtle connection between aging and TLR2 in *S. aureus*–induced bacteremia, which is summarized in Figure 7. In short, TLR2 deficiency and aging enhanced the susceptibility to *S. aureus* bacteremia in an additive manner, as TLR2^−/−^/old mice had the most severe infection, with the highest mortality rate, weight loss, and bacterial load in kidneys. Interestingly, the increased mortality risk associated with *S. aureus* bacteremia is more controlled by aging, whereas other clinical parameters, such as weight loss and kidney abscesses, are more TLR2 dependent. Both aging and TLR2 deficiency impair the immune responses to *S. aureus* bacteremia, such as splenomegaly and cytokine/chemokine production.

## Supplementary Data

Supplementary materials are available at *The Journal of Infectious Diseases* online. Consisting of data provided by the authors to benefit the reader, the posted materials are not copyedited and are the sole responsibility of the authors, so questions or comments should be addressed to the corresponding author.

## Supplementary Material

jiad046_Supplementary_Data

## Notes

***Acknowledgments.*** The authors thank Michelle Schultz for her constructive criticism of the manuscript.

***Author contributions.*** Z. H., Y. F., C. E., R. P., M. M., and T. J. conceived and planned the experiments. Z. H., P. K. K., M. D., A. J., P. G., A. A., and M. M. carried out the experiments. Z. H. and T. J. wrote the manuscript. All authors helped interpret the results, provided critical feedback, and helped shape the research, analysis, and manuscript. All authors contributed to the article and approved the submitted version.

***Disclaimer.*** The funders had no role in the study design, data collection and analysis, decision to publish, or preparation of the manuscript.

***Financial support***. This work was supported by the Swedish Medical Research Council (grants 523-2013-2750 and 2019-01135 to T. J. and 2019-01852 to C. E.); ALF grants from the Swedish state under the agreement between the Swedish government and the county councils, the ALF-agreement (ange anslagsnummer, grants ALFGBG-823941, ALFGBG-933787 and ALFGBG-965074 to T. J., ALFGBG-770411 to A. J., ALFGBG-926621 to R. P., and ALFGBG-965726 to C. E.); E and K.G. Lennanders Scholarship Foundation (A. A. and M. M.); Rune och Ulla Amlövs Stiftelse för Neurologisk och Reumatologisk Forskning (P. K. K., A. A., M. M., and T. J.); Sahlgrenska University Hospitals Research Foundations (A. J. and M. M.); Inger Bendix Foundation for Medical Research (M. M.); Royal Swedish Academy of Sciences (grant ME2015-0119 to A. A.); Magnus Bergvalls Stiftelse (grants 2017-01958 and 2018-02797 to A. A.); Stiftelserna Wilhelm och Martina Lundgrens (P. K. K.); and National Natural Science Foundation of China (grant 81460334 to Y. F.).
