# Supplementary material for: The Impact of Aging and Toll-like Receptor 2 Deficiency on the Clinical Outcomes of Staphylococcus aureus Bacteremia
Source: J Infect Dis. 2023 Feb 20;228(3):332–42. doi: 10.1093/infdis/jiad046 (PMC10420399; doi:10.1093/infdis/jiad046)
Supplement: jiad046_Supplementary_Data [file jiad046_supplementary_data.docx]

**Supplementary information**

**The impact of aging and TLR2 deficiency on the clinical outcomes of *Staphylococcus aureus* bacteremia**

Zhicheng Hu^1,2^*, Pradeep Kumar Kopparapu^1^, Meghshree Deshmukh^1^, Anders Jarneborn^1,3^, Priti Gupta^1^, Abukar Ali^1^, Ying Fei^2^, Cecilia Engdahl^1^, Rille Pullerits^1,4^, Majd Mohammad^1^, Tao Jin^1,3^

* Corresponding author

1. Department of Rheumatology and Inflammation Research, Institute of Medicine, Sahlgrenska Academy, University of Gothenburg, Gothenburg, Sweden

2. Center for Clinical Laboratories, the Affiliated Hospital of Guizhou Medical University, Guiyang, China

3. Department of Rheumatology, Sahlgrenska University Hospital, Gothenburg, Sweden

4. Department of Clinical Immunology and Transfusion Medicine, Sahlgrenska University Hospital, Gothenburg, Sweden.

E-mail: [zhicheng.hu@gu.se](mailto:zhicheng.hu@gu.se)

**Running title: Aging and TLR2 deficiency in *S. aureus* bacteremia**


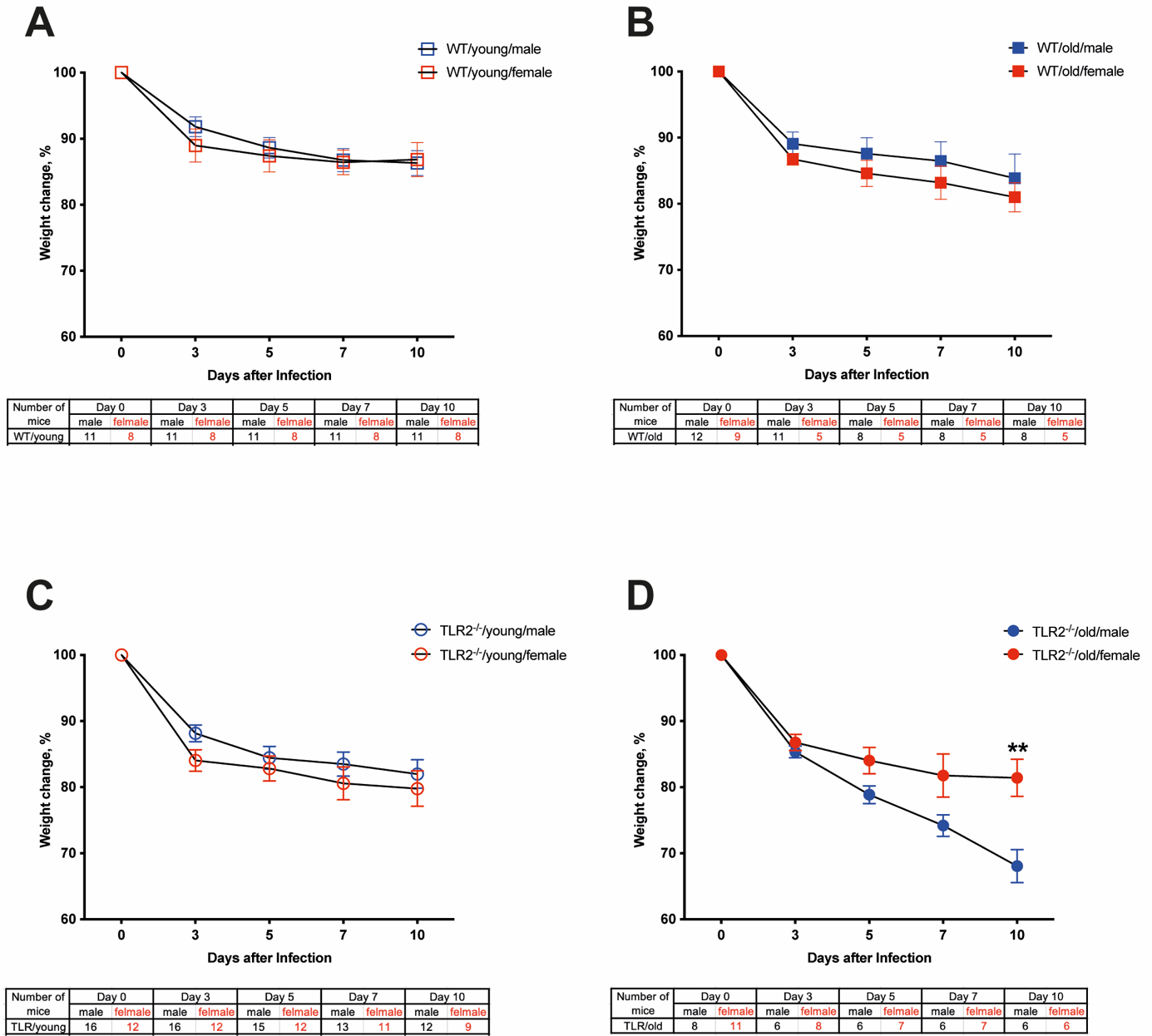


**sfig. 1 The impact of gender difference on weight loss in mice with *S. aureus* bacteremia.** C57BL/6 wild-type (WT) mice and Toll-like receptor 2-deficient (TLR2^-/-^) mice of both sexes, aged from 13–28 weeks (young) and 73–89 weeks (old), respectively, were intravenously inoculated with the *S. aureus* Newman strain at a dose of 1.5x10^6^ CFU/mouse. The changes in percentage of body weight were monitored up to 10 days post-infection. **(A)** WT/young, **(B)** WT/old, **(C)** TLR2^-/-^/young, **(D)** TLR2^-/-^/old. The data were pooled from 2 independent experiments. Statistical evaluations were performed using the Mann-Whitney U test. Data are presented as the mean ± SEM. ***P* <0.01.


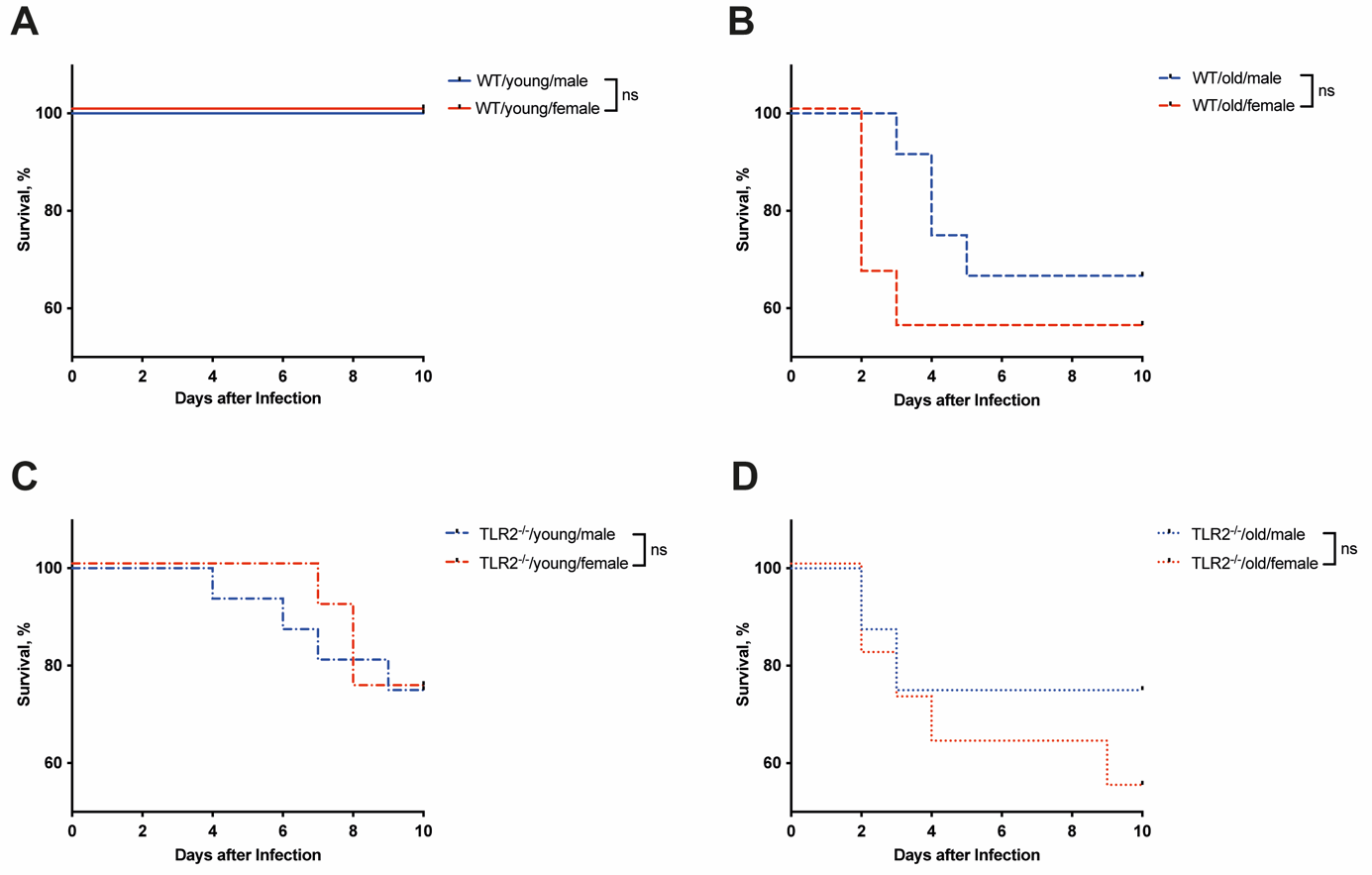


**sfig. 2 Gender has no impact on the mortality of *S. aureus* bacteremia.** C57BL/6 wild-type (WT) mice and Toll-like receptor 2-deficient (TLR2^-/-^) mice of both sexes, aged from 13–28 weeks (young) and 73–89 weeks (old), respectively, were intravenously inoculated with the *S. aureus* Newman strain at a dose of 1.5x10^6^ CFU/mouse. The cumulative survival of the mice was followed every 12 hours for 10 days post-infection. **(A)** WT/young, **(B)** WT/old, **(C)** TLR2^-/-^/young, **(D)** TLR2^-/-^/old. The data were pooled from 2 independent experiments. Statistical evaluations were performed using the log-rank (Mantel-cox) test. ns = not significant.


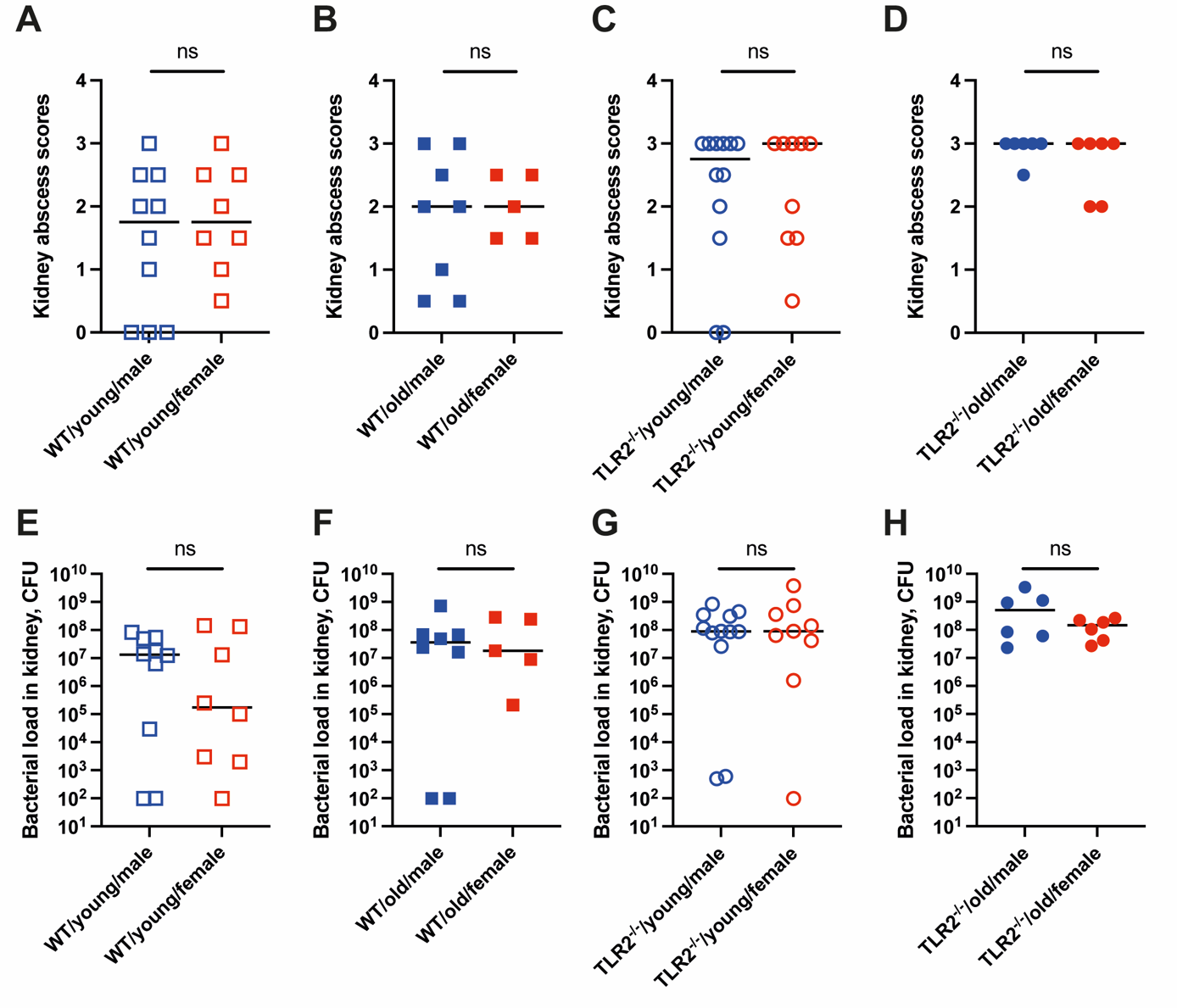


**sfig. 3 Gender has no impact on the bacterial clearance in kidneys.** C57BL/6 wild-type (WT) mice and Toll-like receptor 2-deficient (TLR2^-/-^) mice of both sexes, aged from 13–28 weeks (young) and 73–89 weeks (old): WT/young (n=19), WT/old (n=21), TLR2^-/-^/young (n=28) and TLR2^-/-^/old (n=19), respectively, were intravenously inoculated with the *S. aureus* Newman strain at a dose of 1.5x10^6^ CFU/mouse. The surviving mice were euthanized on day 10 post-infection. **(A-D)** Kidney abscess scores and **(E-H)** persistence of *S. aureus* in kidneys 10 days after infection. The data were pooled from 2 independent experiments. Statistical evaluations were performed using the Mann-Whitney *U* test. Data are presented as the median. ns = not significant.

**sfig. 4** **Age and TLR2 have no impact on the severity of *S. aureus*-induced septic arthritis.** C57BL/6 wild-type (WT) mice and Toll-like receptor 2-deficient (TLR2^-/-^) mice of both sexes, aged from 13–28 weeks (young) and 73–89 weeks (old), respectively, were intravenously inoculated with the *S. aureus* Newman strain at a dose of 1.5x10^6^ CFU/mouse. The surviving mice were euthanized on day 10 post-infection. **(A)** The arthritis severity, **(B)** frequency of clinical arthritis. The data were pooled from 2 independent experiments. Statistical evaluations were performed using two-way ANOVA with Tukey's test **(A)** or Fisher’s exact test **(B)**. Data are presented as the mean ± SEM **(A)**.

**sfig. 5** **Joint destruction was not dependent on TLR2 or age in a murine model of staphylococcal septic arthritis.** C57BL/6 wild-type (WT) mice and Toll-like receptor 2-deficient (TLR2^-/-^) mice of both sexes, aged from 14–17 weeks (young) and 74–76 weeks (old), respectively, were intravenously inoculated with the *S. aureus* Newman strain at a dose of 1.5x10^6^ CFU/mouse. Mice were euthanized and joints were collected for microcomputed tomography (µCT) scan analyses on day 10 post-infection. **(A)** The cumulative bone destruction scores and **(B)** frequency of bone destructions of the joints from all 4 limbs of the mice. Representative µCT scan images **(C**–**F)** showing both intact (left) and heavily eroded (right) joints, **(C)** wrists, **(D)** shoulders, **(E)** hips, and **(F)** knees. Arrows indicate the bone erosion. Statistical evaluations were performed using one-way ANOVA with Tukey's test **(A)** or Fisher’s exact test **(B)**. Data are presented as the mean with SEM **(A)**.

******sfig. 6 TLR2 and aging had no impact on the activation of mouse neutrophil NADPH-oxidase release.** Isoluminol-amplified chemiluminescence was used to measure NADPH oxidase–mediated superoxide anion release by mouse neutrophils. Neutrophils were isolated from the bone marrow of C57BL/6 wildtype (WT) and Toll-like receptor 2-deficient (TLR2^-/-^) mouse of both sexes, aged from 16–17 weeks (young) and 78–83 weeks (old) (n = 2/group). The cells were preincubated at 37 °C for 5 min before being challenged with **(A)** *N*-formyl-Met-Ile-Phe-Leu (fMIFL, 100nM, arrow to the left); **(C)** phenol-soluble modulin (PSM) α3 (500nM); **(E)** phorbol 12-myristate 13-acetate (PMA, 50nM), and measurement of superoxide anion release (O_2_^−^, *y*-axis) over time (min, *x*-axis) from one representative experiment is shown. The peak O_2_^−^ release values of respective stimuli: **(B)** fMIFL, **(D)** PSMα3 and **(F)** PMA were compared, and data are presented as the median.

Supplementary Table 1

Mice used in the *S. aureus* bacteremia experiments.

|  | WT/young | WT/old | TLR2^-/-^/young | TLR2^-/-^/old |
| --- | --- | --- | --- | --- |
| Male | 11 | 12 | 16 | 8 |
| Female | 8 | 9 | 12 | 11 |
| Total | 19 | 21 | 28 | 19 |

Supplementary Table 2

Antibodies used in the FACS panel

| Antibody | Fluorochrome | Clone | Supplier |
| --- | --- | --- | --- |
| CD11b | V450 | M1/70 | BD Horizon™ |
| CD19 | PE | 6D5 | BioLegend® |
| CD335 | PerCP-Cy 5.5 | 29A1.4 | BD Pharmingen™ |
| CD4 | FITC | RM4-5 | BD Pharmingen™ |
| Ly-6C | BV605 | AL-21 | BD Horizon™ |
| Ly-6G | PE-Cy7 | 1A8 | BD Pharmingen™ |
| CD8a | APC-R700 | 53-6.7 | BD Horizon™ |

Supplementary Table 3

Mouse spleen weight (Healthy controls, n=7/group)

| Mouse | weight (g) ** (*p=*0.002) | | | |
| --- | --- | --- | --- | --- |
|  | WT/young | WT/old ☆ | TLR2^-/-^/young | TLR2^-/-^/old ★ |
| 1 | 0.078 | 0.101 | 0.078 | 0.08 |
| 2 | 0.069 | 0.107 | 0.071 | 0.088 |
| 3 | 0.091 | 0.091 | 0.077 | 0.079 |
| 4 | 0.072 | 0.073 | 0.063 | 0.079 |
| 5 | 0.076 | 0.078 | 0.069 | 0.081 |
| 6 | 0.072 | 0.085 | 0.07 | 0.092 |
| 7 | 0.068 | 0.08 | 0.062 | 0.085 |
| 25% Percentile | 0.069 | 0.078 | 0.063 | 0.079 |
| Median | 0.072 | 0.085 | 0.070 | 0.081 |
| 75% Percentile | 0.078 | 0.101 | 0.077 | 0.088 |
| Mean | 0.075 | 0.088 | 0.070 | 0.083 |
| Std. Deviation | 0.008 | 0.013 | 0.006 | 0.005 |
| Std. Error of Mean | 0.003 | 0.005 | 0.002 | 0.002 |

The data were analyzed by one-way ANOVA with Tukey's test: ☆ WT/young vs. WT/old (*); ★ TLR2^-/-^/young vs. TLR2^-/-^/old (*).

Supplementary Table 4

Mouse serum cytokine levels (Healthy controls, n=4/group)

| Mouse | IL-6 (pg/ml) | | | | KC (pg/ml) | | | |
| --- | --- | --- | --- | --- | --- | --- | --- | --- |
|  | WT/young | WT/old | TLR2^-/-^/young | TLR2^-/-^/old | WT/young | WT/old | TLR2^-/-^/young | TLR2^-/-^/old |
| 1 | 2.929 | 1.916 | 1.721 | 2.662 | 99.214 | 224.689 | 117.846 | 127.960 |
| 2 | 2.839 | 3.229 | 2.750 | 1.531 | 163.260 | 187.388 | 198.224 | 123.894 |
| 3 | 2.603 | 2.000 | 2.662 | 2.810 | 208.015 | 186.170 | 143.286 | 348.930 |
| 4 | 3.199 | 2.810 | 1.667 | 3.351 | 146.984 | 236.455 | 115.986 | 240.830 |
| 25% Percentile | 2.662 | 1.937 | 1.681 | 1.814 | 111.2 | 186.5 | 116.5 | 124.9 |
| Median | 2.884 | 2.405 | 2.192 | 2.736 | 155.1 | 206.0 | 130.6 | 184.4 |
| 75% Percentile | 3.132 | 3.124 | 2.728 | 3.216 | 196.8 | 233.5 | 184.5 | 321.9 |
| Mean | 2.893 | 2.489 | 2.200 | 2.589 | 154.4 | 208.7 | 143.8 | 210.4 |
| Std. Deviation | 0.246 | 0.637 | 0.586 | 0.765 | 44.92 | 25.74 | 38.34 | 107.1 |
| Std. Error of Mean | 0.123 | 0.319 | 0.293 | 0.382 | 22.46 | 12.87 | 19.17 | 53.54 |

The data were analyzed by one-way ANOVA with Tukey's test.

Supplementary Table 5

Mouse peripheral blood leukocyte frequency by flow cytometry (Healthy controls, n=4/group)

| Mouse | Neutrophils (%) * (*p=*0.03) | | | | Macrophages (%) (*p=*0.05) | | | |
| --- | --- | --- | --- | --- | --- | --- | --- | --- |
|  | WT/young | WT/old | TLR2^-/-^/young | TLR2^-/-^/old | WT/young | WT/old | TLR2^-/-^/young | TLR2^-/-^/old |
| 1 | 4.57 | 4.05 | 1.98 | 0.28 | 3.44 | 1.98 | 2.18 | 0.27 |
| 2 | 3.82 | 1.14 | 2.41 | 1.39 | 4.28 | 1.26 | 4.15 | 1.97 |
| 3 | 2.47 | 3.39 | 0.96 | 0.29 | 4.83 | 5.53 | 1.56 | 0.22 |
| 4 | 3.66 | 3.54 | 3.82 | 2.10 | 5.65 | 3.43 | 4.89 | 2.70 |
| 25% Percentile | 2.77 | 1.70 | 1.22 | 0.283 | 3.65 | 1.44 | 1.72 | 0.233 |
| Median | 3.74 | 3.47 | 2.20 | 0.840 | 4.56 | 2.71 | 3.17 | 1.12 |
| 75% Percentile | 4.38 | 3.92 | 3.47 | 1.92 | 5.45 | 5.01 | 4.71 | 2.52 |
| Mean | 3.63 | 3.03 | 2.29 | 1.02 | 4.55 | 3.05 | 3.20 | 1.29 |
| Std. Deviation | 0.869 | 1.29 | 1.19 | 0.891 | 0.930 | 1.88 | 1.58 | 1.24 |
| Std. Error of Mean | 0.435 | 0.646 | 0.593 | 0.446 | 0.465 | 0.942 | 0.790 | 0.622 |

|  | CD4+ T cells (%) * (*p=*0.03) | | | | CD8+ T cells (%) ***(K-W, *p=*0.0009) | | | |
| --- | --- | --- | --- | --- | --- | --- | --- | --- |
| Mouse | WT/young | WT/old | TLR2^-/-^/young | TLR2^-/-^/old | WT/young | WT/old | TLR2^-/-^/young | TLR2^-/-^/old |
| 1 | 3.90 | 1.71 | 3.70 | 0.14 | 9.74 | 1.59 | 5.84 | 0.14 |
| 2 | 5.37 | 0.12 | 1.22 | 1.47 | 4.77 | 0.36 | 6.40 | 2.08 |
| 3 | 2.77 | 3.57 | 1.78 | 0.04 | 8.91 | 2.19 | 5.37 | 1.09 |
| 4 | 3.34 | 1.93 | 0.40 | 1.92 | 15.98 | 4.43 | 15.85 | 4.46 |
| 25% Percentile | 2.91 | 0.518 | 0.605 | 0.0650 | 5.81 | 0.668 | 5.49 | 0.378 |
| Median | 3.62 | 1.82 | 1.50 | 0.805 | 9.33 | 1.89 | 6.12 | 1.59 |
| 75% Percentile | 5.00 | 3.16 | 3.22 | 1.81 | 14.4 | 3.87 | 13.5 | 3.87 |
| Mean | 3.85 | 1.83 | 1.78 | 0.893 | 9.85 | 2.14 | 8.37 | 1.94 |
| Std. Deviation | 1.12 | 1.41 | 1.40 | 0.946 | 4.63 | 1.70 | 5.01 | 1.86 |
| Std. Error of Mean | 0.558 | 0.706 | 0.701 | 0.473 | 2.31 | 0.852 | 2.50 | 0.928 |

| Mouse | B cells (%) * (*p=*0.01) | | | |
| --- | --- | --- | --- | --- |
|  | WT/young | WT/old | TLR2^-/-^/young | TLR2^-/-^/old |
| 1 | 37.40 | 18.30 | 14.60 | 1.16 |
| 2 | 24.30 | 6.13 | 22.30 | 10.00 |
| 3 | 30.90 | 24.40 | 10.20 | 3.44 |
| 4 | 48.70 | 23.50 | 34.20 | 19.80 |
| 25% Percentile | 26.0 | 9.17 | 11.3 | 1.73 |
| Median | 34.2 | 20.9 | 18.5 | 6.72 |
| 75% Percentile | 45.9 | 24.2 | 31.2 | 17.4 |
| Mean | 35.3 | 18.1 | 20.3 | 8.60 |
| Std. Deviation | 10.4 | 8.41 | 10.5 | 8.35 |
| Std. Error of Mean | 5.20 | 4.20 | 5.26 | 4.18 |

Normal distribution data were analyzed by one-way ANOVA with Tukey's test, Kruskal-Wallis (K-W) test with Dunn's multiple comparisons test was applied for non-normal distribution data.
